# Mitochondrial DAMPs Induce Endotoxin Tolerance in Human Monocytes: An Observation in Patients with Myocardial Infarction

**DOI:** 10.1371/journal.pone.0095073

**Published:** 2014-05-05

**Authors:** Irene Fernández-Ruiz, Francisco Arnalich, Carolina Cubillos-Zapata, Enrique Hernández-Jiménez, Raúl Moreno-González, Víctor Toledano, María Fernández-Velasco, Maria T. Vallejo-Cremades, Laura Esteban-Burgos, Rebeca Pérez de Diego, Miguel A. Llamas-Matias, Elena García-Arumi, Ramón Martí, Lisardo Boscá, Antoni L. Andreu, José Luis López-Sendón, Eduardo López-Collazo

**Affiliations:** 1 Tumor Immunology Lab, IdiPAZ, Hospital La Paz, Madrid, Spain; 2 Innate Immunity Group, IdiPAZ, La Paz Hospital, Madrid, Spain; 3 Internal Medicine Service, Hospital La Paz, Madrid, Spain; 4 Cardiology Service, Hospital La Paz, Madrid, Spain; 5 Institute of Biomedical Research, Alberto Sols, Madrid, Spain; 6 Laboratory of Immunogenetics of Diseases, IdiPAZ, La Paz Hospital, Madrid, Spain; 7 EMPIREO Laboratory, Madrid, Spain; 8 Department of Mitochondrial and Neuromuscular Pathology, Vall d'Hebron Research Institute, Barcelona, Spain; RIKEN Advanced Science Institute, Japan

## Abstract

Monocyte exposure to mitochondrial Danger Associated Molecular Patterns (DAMPs), including mitochondrial DNA (mtDNA), induces a transient state in which these cells are refractory to further endotoxin stimulation. In this context, IRAK-M up-regulation and impaired p65 activity were observed. This phenomenon, termed endotoxin tolerance (ET), is characterized by decreased production of cytokines in response to the pro-inflammatory stimulus. We also show that monocytes isolated from patients with myocardial infarction (MI) exhibited high levels of circulating mtDNA, which correlated with ET status. Moreover, a significant incidence of infection was observed in those patients with a strong tolerant phenotype. The present data extend our current understanding of the implications of endotoxin tolerance. Furthermore, our data suggest that the levels of mitochondrial antigens in plasma, such as plasma mtDNA, should be useful as a marker of increased risk of susceptibility to nosocomial infections in MI and in other pathologies involving tissue damage.

## Introduction

The endotoxin tolerant (ET) state is a clinical phenomenon not restricted to sepsis; it has also been observed in other pathologies such as acute coronary syndromes (ACS), cystic fibrosis and cancer [1]–[5]. While ET has been considered a protective mechanism against septic shock and ischemia, its incidence is also associated with a high risk of secondary infections. This refractory state is associated with the innate immune system and in particular, with monocytes and macrophages, which act as the primary participants. Several authors have classified the activation of monocytes/macrophages (MΦs) into classical (M1) and alternative (M2) categories. Whereas M1 macrophage polarization is characterized by an inflammatory response, the functions of alternatively activated macrophages, or M2, involve the control of inflammatory responses, enhanced phagocytic activity and tissue repair [6]. Numerous studies reveal substantial similarities between M2 polarization and ET states. These studies propose that this phenomenon can be considered another form of alternative activation triggered by bacterial signatures such as lipopolysaccharides (LPS) [7].

We and other authors have shown that tolerant human monocytes are characterized by rapid IRAK-M overexpression, high levels of CD163 and low HLA expression [1], [2]. In-depth studies of ET development in gene-deficient mice analyzed the participation of intracellular molecules in this process and established the roles of SHIP-1, A20 and IRAK-M. This pseudokinase could be considered a “master regulator” of ET because it is consistently induced into ET [8], [9] and is implicated in human pathologies in which ET is manifest, such as sepsis [10], cancer [11], ACS [3] and asthma [12]. In a human *in vitro ET* model, rapid IRAK-M up-regulation was described and is expressed in freshly isolated sepsis monocytes [10]. More importantly, IRAK-M up-regulation was associated with high mortality after Gram-negative-induced sepsis [9].

One of the illnesses in which ET takes place is acute coronary syndrome (ACS). ACS includes a range of thrombotic coronary artery diseases, such as unstable angina (UA), ST-elevation myocardial infarction (STEMI) and non-ST-elevation myocardial infarction (NSTEMI). The innate immune system plays a key role in the progression of atherosclerotic lesions and in remodeling after myocardial infarction (MI) [13], [14]. In this context, the activation of the innate immune response mediated by MΦs releases factors that cause inflammation [15], tissue damage and plaque instability [16]. We have previously reported that the monocytes of ACS patients show a pro-inflammatory phenotype after 1–3 h of MI (STEMI and NSTEMI), with up-regulation of TNF-α [3]. These cells have high levels of IRAK-M, thus providing negative feedback regulation for the pro-inflammatory response. This is a classic paradigm of ET producing a hypo-responsive state following an LPS challenge [10], [17]. These data suggest a potential switch from a pro-inflammatory phenotype or M1 to a tolerant state that matches an M2 phenotype in these cells. The absence of previous infections in these patients suggested the existence of “damps” that trigger a tolerant state. Several internal factors could act as initial stimuli in this respect; molecules known as Danger Associated Molecular Patterns (DAMPs) [18] are candidates, such as hyaluronic acid, High Mobility Group B1 and HSP. Moreover, due to the breakdown of tissue during MI, other DAMPs could spread, such as those from mitochondria. Mitochondria are evolutionary endosymbionts derived from bacteria, and they contain DNA similar to bacterial DNA [19].

In the present study, we analyzed the potential role of mitochondrial DAMPs, particularly mitochondrial DNA (mtDNA), as ET inducers in human monocytes using an *in vitro* model of ET and samples from patients with ACS.

## Materials and Methods

### Patients

The study enrolled 75 consecutive patients with ACS who underwent coronary artery angiography at the Cardiology Service of La Paz University Hospital from March 2010 through July 2010. Forty-five patients had acute MI (21 patients with and 24 patients without ST-segment elevation in the ECG, classified as STEMI and NSTEMI, respectively), and 30 patients suffered from UA. Twenty age- and sex-matched healthy volunteers (HV) without a history of coronary disease or cardiovascular risk factors were included as controls (Table 1). We excluded patients with acute or chronic inflammatory diseases, immune suppression, active acute or chronic infectious diseases, a history of renal or hepatic diseases, poorly controlled diabetes (HbA1_c_ ≥7 g/dl) or malignancies, those who had undergone a surgical procedure in the preceding 3 months and those who were on treatment with immunosuppressive drugs.

**Table 1:** Comparison of demographics and clinical data between groups.

	STEMI (n = 21)	NSTEMI (n = 24)	UA (n = 30)	Controls (n = 20)	P value
**Age, years**	61.3±10.6	62.4±9.6	60.7±8.9	60.4±10.3	ns
**Gender, male, number (%)**	13 (62%)	16 (67%)	19 (63%)	12 (60%)	ns
**Previous history of CAD**	5 (24%)	5 (21%)	2 (7%)		
**Cardiovascular risk factors, n (%)**					
Hypertension, (>140/90 mm Hg)	13 (62)	14 (58%)	11 (37%)	0	<0.05
Diabetes mellitus	12 (57%)	12 (50%)	6 (20%)	0	<0.01
Current smoking	5 (24%)	6 (25%)	6 (20%)	0	ns
Cholesterolemia>5.17 mmol/L	7 (33%)	9 (37%)	8 (27%)	0	ns
Family history of CAD	3 (14%)	4 (17%)	4 (13%)	0	ns
At least 3 risk factors	8 (38%)	8 (33%)	3 (10%)	0	ns
Previous history of CAD	4 (19%)	4 (17%)	2 (7%)	0	ns
**Cardiac catheterization**					
Vessel score	2.1±0.8	1.6±0.8	1.1±0.6	—	<0.05
**Laboratory data and follow-up**					
Leukocyte count (x 10^9^/mL)	13.1±2.48	12.7±2.24	8.8±1.75	7.5±1.46	<0.01
Creatinine (µmol/L)	72.4±15.6	71.5±14.3	70.4±16.2	69.8±14.7	ns
Glucose (mmol/L)	6.4±1.17	6.2±1.02	5.54±0.94	5.30±0.45	<0.05
Troponin I peak level (ng/mL)	84±23	76±25	18±10	<6.0	<0.001
CRP (mg/dl)	14.24±5.09	10.60±2.10	9.54±2.28		<0.001
IL6 (ng/L)	12.33±2.90	10.75±2.20	8.77±2.09		<0.001
BNP (pg/ml)	482±255	364±191	58±37		<0.001
**Infectious episodes within 2 months after discharge**	4 (19%)	3 (12%)	0		<0.01
**Fatal MI during the first year**	1 (5%)	1 (4%)	0		ns

Data are Mean±SD, IQR or number (%). STEMI or NSTEMI *vs.* UA patients (ANOVA/Dunn). ns: non-significant; CRP: C-reactive protein; IQR: interquartile range; BNP: Brain natriuretic peptide.

The sample size was estimated based on our previous study [3]. In addition, a multivariate logistic regression analysis was performed to evaluate the effects of gender; the presence of CV risk factors (arterial hypertension, diabetes, active smoking, hypercholesterolemia, obesity), total leukocyte count, serum concentrations of glucose, creatinine, and C-reactive protein, length of hospital stay, and use of a urinary bladder catheter, on the development of ET state or the appearance of infections in the 30 days following hospital discharge.

The patients with UA [20], NSTEMI and STEMI were diagnosed according to previous reports (Supplementary Methods in Data S1). Sixty milliliters of blood were taken from a peripheral vein after the first 30 h (36±6 h) of hospital admission to avoid the initial acute pro-inflammatory response to ischemic injury [3]. The participants were white Spanish residents. The study protocol adhered to the ethical guidelines of the 1975 Declaration of Helsinki and received approval from the Ethics Committee of La Paz Hospital in Madrid. All participants provided their written informed consent to participate in the study and the Ethic Committee approved this consent procedure.

### Follow-up study

The follow-up information was obtained at prospectively determined monthly visits to our outpatient clinic, from discharge reports in case of emergency admission, and from telephone interviews with patients, their closest relative, or their referring physician.

### Reagents

The following antibodies were used: anti-CD14-APC, anti-MHCII-DQ-PE, anti-MHCII-DR-APC (Immunostep, Salamanca, Spain) and anti-CD163-PE (Miltenyi Biotec, Auburn, CA, USA). The medium used for the cell culture was DMEM from Invitrogen. The LPS from *Salmonella abortus* was a kind gift from Dr. Galanos (Max Planck Institute of Immunobiology and Epigenetics, Freiburg, Germany).

### Mitochondria Isolation

The mitochondria were isolated as described [21], with minor modifications. Briefly, exponentially growing HeLa cells were harvested, washed twice with 1 mMTris–HCl (pH 7.0), 0.13 M NaCl, 5 mMKCl, 7.5 mM MgCl_2_, and broken in one-half of the packed cell volume of 3.5 mMTris–HCl (pH 7.8), 2 mMNaCl, 0.5 mM MgCl_2_, by using a Thomas homogenizer with a motor-driven Teflon pestle. The homogenate was immediately mixed with one-ninth of the cell volume of 0.35 M Tris–HCl (pH 7.8), 0.2 M NaCl, 50 mM MgCl_2_, and spun for 3 min at 1600 g to pellet unbroken cells, debris and nuclei. The supernatant was re-centrifuged under the same conditions. The final supernatant was spun at 13,000 g for 2 minutes. The mitochondrial pellets were washed serially with 35 mMTris–HCl (pH 7.8), 20 mMNaCl, 5 mM MgCl_2_, then twice with 10 mMTris–HCl (pH 7.4), 1 mM EDTA, 0.32 M sucrose, and resuspended in the appropriate buffer. The entire purification process was performed at 4°C. All these preparations were endotoxin free (tested by the LAL Endotoxin Assay Kit).

### Mitochondrial DNA from Clinical Material

The primers for the real-time PCR analysis of mtDNA were: 5′CCACGGGAAACAGCAGTGAT3′ and 5′CTATTGACTTGGGTTAATCGTGTGA3′. The TaqMan probe (6FAM-5′TGCCAGCCACCGCG3′-MGB) was labeled at the 5′ end with a fluorescent reporter, 6FAM.

The 20 µL PCR reaction contained 1× TaqMan Universal PCR Master Mix (Applied Biosystems P/N 4304437), 112 nM of each mtDNA primer, 125 nM of mtDNATaqMan probe and 5 µL DNA extract. The PCR conditions were 2 min at 50°C and 10 min at 95°C, followed by 40 cycles 15 s of denaturation at 95°C and 60 s of annealing/extension at 60°C. Calibration curves were used to quantify mtDNA as described previously [22].

### Preparation of Mitochondrial Lysates, Mitochondrial DNA and Nuclear DNA

Isolated mitochondrial pellets were resuspended in 500 µl of 20 mM Hepes-NaOH, (pH 7.4) 75 mMNaCl, 50 mM EDTA. A protease inhibitor cocktail (4∶100) was added to the suspension. The disrupted mitochondrial suspension was centrifuged at 13,000 g for 10 min at 4°C. The supernatants were used for the experiments. The protein concentrations of the mitochondrial lysate solutions were determined by the BCA Protein Assay (Pierce). Mitochondrial DNA was extracted from the isolated mitochondria and the nuclear DNA was extracted from the isolated nuclei using the QIAampDNA Mini kit (Qiagen). Mitochondrial and nuclear DNA concentrations were determined with a NanoDrop Spectrophometer (Thermo Scientific). No protein contamination was found. The nuclear DNA in the mtDNA extract was less than 0.1%, as measured by quantitative PCR as described above. The mitochondrial lysate and the mitochondrial and nuclear DNA were prepared under sterile conditions. All the extracts were endotoxin-free, as assayed with the Sigma E-Toxate reagent from *Limulus polyphemus* (Sigma).

### Flow Cytometer Analysis

For surface marker staining, the cells were labeled with the following monoclonal antibodies: allophycocyanin (APC)-conjugated anti-human CD14, phycoerythrin (PE) conjugated anti-human HLA-DQ and allophycocyanin (APC)-conjugated anti-human HLA-DR (all from Immunostep, Spain); and PE-conjugated anti-human CD163 (Miltenyi Biotec, USA). Matched isotype antibodies were used as negative controls. The cells were incubated for 30 min at 4°C in the dark. The data was analyzed by flow cytometry using a BD FACSCalibur flow cytometer (BD Biosciences, USA). Data was analyzed with FlowJo software (Tristar, USA).

### Monocyte isolation from PBMC

The peripheral blood mononuclear cells (PBMC) were isolated from the buffy coats [4]. The monocytes were obtained by adherence, as previously described [4]. The purity of the monocyte cultures was tested by CD14 labeling and flow cytometry analysis (average 89% of CD14-positive cells). Other cell surface markers were also tested (CD16b: 4.2%; CD1a: 3.8%; CD89: 91%, data not shown). The same protocol was used to obtain monocytes from all patients. All reagents used for cell cultures were endotoxin-free, as assayed with the *Limulus* amebocyte lysate test (Cambrex). The laboratory workers did not know any details about the samples from patients at processing time. In order to avoid circadian variability in cytokine production, all the samples were drawn during the morning (from 8 to 11 AM). Moreover, blood samples for laboratory measurements were collected immediately before coronary angiography and interventions.

### Cytometric Bead Array (CBA) and Flow Cytometer Analysis

The cytokine levels in the culture supernatants were determined using the CBA Flex Set (BD Biosciences, USA) following the manufacturer's protocol. For surface marker staining, the cells were labeled with the indicated monoclonal antibodies (Supplementary Methods in Data S1) and analyzed by flow cytometry using a BD FACSCalibur flow cytometer (BD Biosciences, USA). The data were analyzed with FlowJo software (Tristar, USA).

### Immunofluorescence

The cells were fixed in 2% buffered paraformaldehyde at room temperature (RT) for 15 min, then permeabilized with methanol for 10 min, followed by two washes in phosphate-buffered saline (PBS). The samples were blocked by incubation for 60 min at RT with PBS containing 3% bovine serum albumin. The samples were incubated overnight at 4°C with anti-p65 (Santa Cruz, CA), followed by three 5-min washes with PBS. The bound primary antibody was detected by incubation for 45 min with the goat secondary antibody conjugated to Alexa 594 (dil. 1/1000, Molecular Probes) for 30 min at RT. The slides were washed three times in PBS and mounted under a coverslip using VectaShield mounting medium with DAPI (Vector Laboratories, Inc). All samples were visualized by confocal microscopy, which was performed on a Leica SP5 using a 40× dry objective lens. An emission/excitation laser for DAPI and Alexa 594 was used. A sample with no primary antibody was always included to control for background generated by the secondary Abs. The image processing was performed using PHOTOSHOP CS software (Adobe Systems, San Jose, CA).

### RNA Isolation and Quantification

RNA isolation and quantification were performed as previously described [10].

### M1/M2 Score

The M1/M2 score was calculated using mRNA expression levels of TNFα and IL10 in Mφ from patients with ACS and HV at baseline and after 3 h of LPS stimulation [23]. At baseline, an M1 phenotype was classified as [TNFα]>[TNFα Mean+SD of HV] and [IL10]<[ IL10 Mean-SD of HV]; M2 was classified as [TNFα]<[TNFα Mean-SD of HV] and [IL10]>[IL10 Mean+SD of HV]; and M0 was chosen when levels of both cytokines were in the range of Mean±SD of HV. Mφ polarization is a continuum, thus two intermediate states were considered: M1/M0 ([TNFα] >[TNFα Mean+SD of HV] and [IL10] = [IL10 Mean±SD of HV]); and M0/M2 ([TNFα]  = [ TNFαMean±SD of HV] and [IL10]>[ IL10 Mean+SD of HV]).

After LPS exposure, there is no M0 state, as the HV would have a classic pro-inflammatory M1 response. Therefore, M1 groups all patients with mRNA levels of both cytokines in the range of Mean±SD of HV after LPS stimulation. M1 high was assigned when [TNFα] >[TNFα Mean+SD of the HV] and [IL10]<[IL10 Mean-SD of the HV] and M2 when [TNFα] <[TNFα Mean-SD of the HV] and [IL10]>[IL10 Mean+SD of the HV].

### Western blot analysis

The mitochondrial lysates were analyzed by Western blot as previously described [3] using Lamin B, VDAC1/2/3, IRAK-M and Actin antibodies (Santa Cruz, CA).

### Statistical Analysis

The number of experiments analyzed is indicated in each Figure. The data were collected from a minimum of three experiments and are expressed as Mean±SD. The statistical significance was calculated using a one-way ANOVA/Dunnet analysis or an unpaired t-test, where appropriate. The correlations were assessed with Spearman's rank correlation for non-normally distributed data. The differences were considered significant at p<0.05, using Prism 5.0 software (GraphPad, USA).

## Results

### Mitochondrial DAMPs induce an endotoxin tolerance state in human monocytes

**Figure 1:**
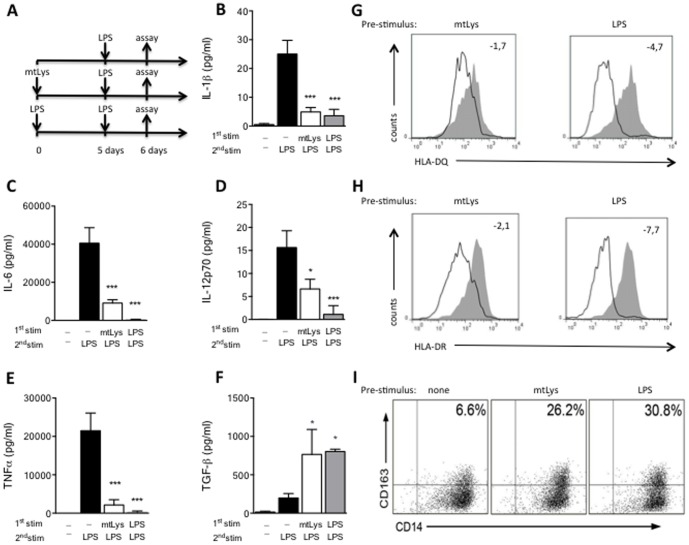
Modulation of human monocytes by exposure to mitochondrial DAMPs. (**A**) Workflow diagram: MΦs from HV were pre-exposed to mitochondrial DAMPs (mtLys, 20 µg/ml, white bars), LPS (10 ng/ml, gray bars) or left untreated (black bars) for 5 days, then stimulated for 24 h with LPS, as described in the scheme; **B–F**: (**B**) Protein levels of IL1β, (**C**) IL6, (**D**) IL12p70, (**E**) TNFα and (**F**) TGF-β in culture supernatants (measured by CBA). Data are shown as Mean±SD, n = 3; *p<0.01, ***p<0.001; ns: non-significant *vs.*corresponding control of untreated 5d +LPS stimulus (ANOVA/Dunn). (**G and H**): Histogram plots of surface HLA-DQ (up) and HLA-DR (down) expression on CD14+ MΦs evaluated by flow cytometry, after 24 h of LPS challenge in MΦs pre-exposed 5 days to mtLys (left panels) or LPS (right panels), compared to MΦs with no pre-treatment (gray filled, all panels). Mean Fold decrease of MFI compared to corresponding control with no pre-treatment is shown. (**I**) CD163+CD14+ frequencies in MΦs pre-exposed 5 days to mtLys (**central panel**), LPS (**right panel**) or medium (**left panel**). Representative dot plots of three independently performed experiments are shown. Values in the upper-left quadrants indicate the Mean frequencies of CD163+CD14+ subsets.

**Figure 2:**
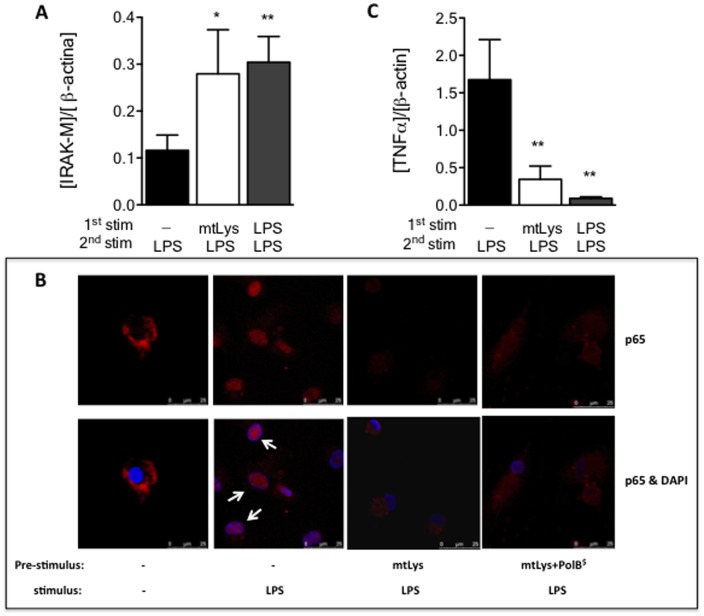
Mitochondrial DAMPs impaired NFκB activation. MΦs from HV were pre-exposed to mitochondrial DAMPs (mtLys, 20 µg/ml, white bars), LPS (10 ng/ml, gray bars) or left untreated (black bars) for 5 days, then stimulated for 1 h with LPS. The qPCR analyses of (**A**) IRAK-M mRNA and (**C**) TNFα levels were performed. The ratio of mRNA [gene]/[β-*actin*] is given. Data are Mean ± SD. *p<0.05, **p<0.01 *vs.* corresponding control with no pre-treatment (ANOVA/Dunn). (**B**) Confocal microscopy analysis of MΦs under conditions indicated; representative pictures of three independently performed experiments are shown. The arrows indicate p65 in the nucleus. ^$^Polymyxyn B (2 µl/ml) was added to mtLys.

From our previous studies, we have observed that human MΦs pre-conditioned with endotoxins, such as LPS, fail to orchestrate a classic inflammatory response when they are challenged with LPS [1], [4], [10], [24]. Several authors identified this tolerant state with an M2 phenotype. To study the potential role of mitochondrial DAMPs as an M2 phenotype inductor, human monocytes were exposed for 5 days to mitochondrial lysate (mtLys) obtained from the HeLa cell line. Next, cultures were challenged with LPS (see scheme in Fig. 1A). As with LPS pre-stimulation, mtLys impeded the production of inflammatory cytokines in a second stimulation with LPS (Fig. 1B–1E). In contrast, a patent TGF-β induction was observed in both LPS and mtLys pretreatment (Fig. 1F) [25]. Other features of ET, such as HLA down-regulation and CD163 expression, were also observed (Fig. 1G-1I and Table S1). Moreover, the levels of the negative regulator IRAK-M increased significantly when MΦs were pre-exposed to mtLys (Fig. 2A and Fig. S1). Subsequently, p65 translocation into the nucleus was inhibited (Figure 2B); this process impaired the expression of several cytokines such as TNFα (Fig. 1B–1E and Fig. 2C). The p65 inhibition was also observed in the presence of the endotoxin-recruited antibiotic Polymyxin B (Fig. 2B, last panel) and it was confirmed by an ELISA assay (data not shown). Note that the purity of the mtLys was assessed by a Western blot analysis (Fig. S2) and an LAL test.

### Mitochondrial DNA induces an M2 phenotype in human monocytes

**Figure 3:**
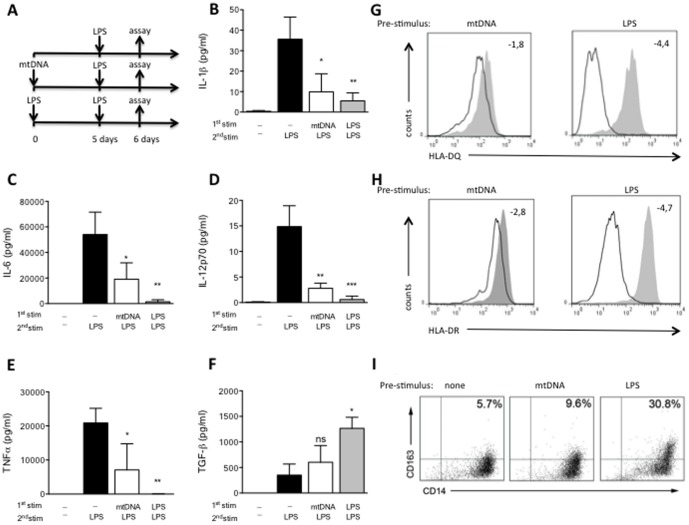
Modulation of human monocytes by exposure to mitochondrial DNA. (**A**) Workflow diagram: MΦs from HV were pre-exposed to mitochondrial DNA isolated from HeLa (mtDNA, 5 µg/ml, white bars), LPS (10 ng/ml, gray bars) or left untreated (black bars) for 5 days, then stimulated for 24 h with LPS, as described in the scheme. **B–F**: (**B**) Protein levels of IL1β, (**C**) IL6, (**D**) IL12p70, (**E**) TNFα and (**F**) TGF-β in culture supernatants (measured by CBA). Data are shown as Mean±SD, n = 3; *p<0.05,**p<0.01 ***p<0.001; ns: non-significant *vs.* corresponding control of untreated 5d +LPS stimulus (ANOVA/Dunn). (**G and H**): Histogram plots of surface HLA-DQ (up) and HLA-DR (down) expression on CD14+ MΦs evaluated by flow cytometry, after 24 h of LPS challenge in MΦs pre-exposed 5 days to mtDNA (left panels) or LPS (right panels), compared to MΦs with no pre-treatment (gray filled, all panels). Mean Fold decrease of MFI compared to corresponding control with no pre-treatment is shown. (**I**) CD163+CD14+ frequencies in MΦs pre-exposed 5 days to mtDNA (**central panel**), LPS (**right panel**) or medium (**left panel**). Representative dot plots of three independently performed experiments are shown. Values in the upper-left quadrants indicate the Mean frequencies of CD163+CD14+ subsets.

**Figure 4:**
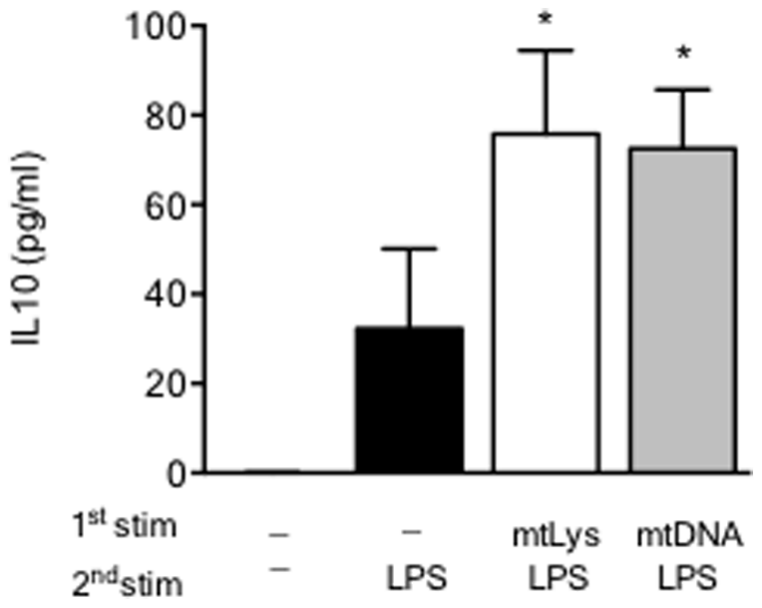
Modulation of IL10 production after exposure to mitochondrial DAMPs and mitochondrial DNA. MΦs from HV were pre-exposed to both mitochondrial DAMPs (mtLys, 20 µg/ml, white bars) and mitochondrial DNA isolated from HeLa (mtDNA, 5 µg/ml, white bars) or left untreated (black bars) for 5 days, then stimulated for 24 h with LPS, as described in the scheme of Figure 1 and Figure 3. Protein levels of IL10 in culture supernatants (measured by CBA) is given. Data are shown as Mean±SD, n = 3; *p<0.01 *vs.* corresponding control of untreated 5d+LPS stimulus (ANOVA/Dunn).

In line with our findings with mtLys, prolonged mitochondrial DNA (mtDNA) pretreatment reduced the inflammatory response in human MΦs challenged with LPS. Again the production of IL-1β, IL-6, IL-12p70 and TNFα were impaired (Fig. 3A–3E), while the anti-inflammatory cytokine TGFβ was slightly up-regulated (Fig. 3F). Similar results were obtained when HLA-DR, HLA-DQ and CD163 were analyzed (Fig. 3G–3I and Table S2). Curiously, the observed M2 phenotype was not completely reproduced when nuclear DNA rather than mtDNA was used (Fig. S3 and Table S3). Note that both mtLys and mtDNA pretreatment increased IL10 production after the LPS challenge (Fig. 4).

### Mitochondrial DNA (mtDNA) levels in plasma are high in patients with multiple coronary lesions and these levels correlate with tolerance

Having established that, after mitochondrial DAMPs, human MΦs are locked in a refractory state, we then analyzed the levels of mtDNA in the plasma of patients who had suffered from coronary lesions. We demonstrated in a previous study that patients with ACS have significantly increased levels of IRAK-M, suggesting a potential refractoriness of their circulating cells. Therefore, 75 ACS patients and 20 age- and sex-matched healthy volunteers (HV) were enrolled (see Table 1). Forty-five patients had acute myocardial infarction (MI): 21 patients with, and 24 patients without ST-segment elevation in the ECG (STEMI and NSTEMI, respectively); and 30 patients suffered from Unstable Angina (UA). See Table 1 and the Supplementary material for details.

**Figure 5:**
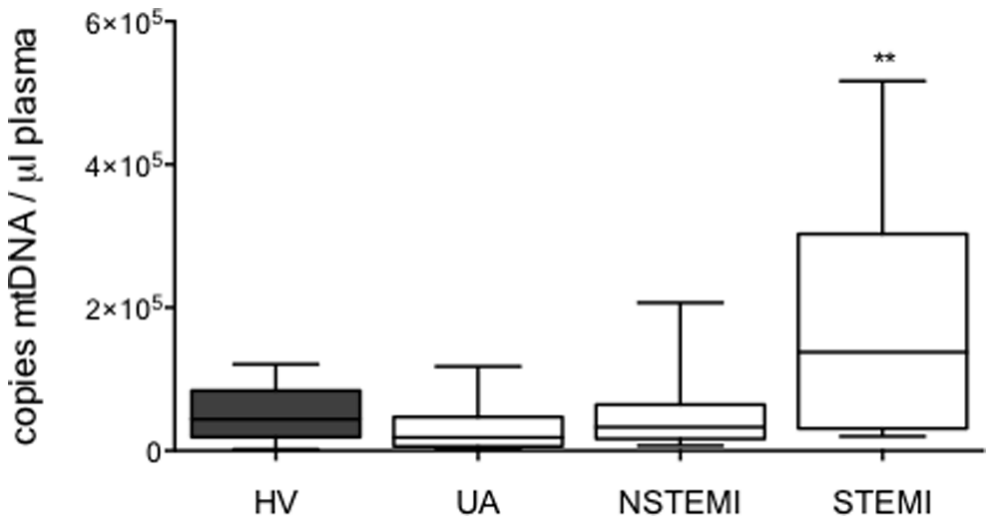
Mitochondrial DNA levels in plasma are increased in STEMI patients. Plasma mitochondrial DNA (mtDNA) levels of ACS patients (white bars) and HV (gray bar). Data are shown as box plots with Mean±SD. HV n = 20, patients with ACS: UA n = 30, NSTEMI n = 24, STEMI n = 21; **p<0.01, patients *vs.* HV (ANOVA/Dunn).

**Figure 6:**
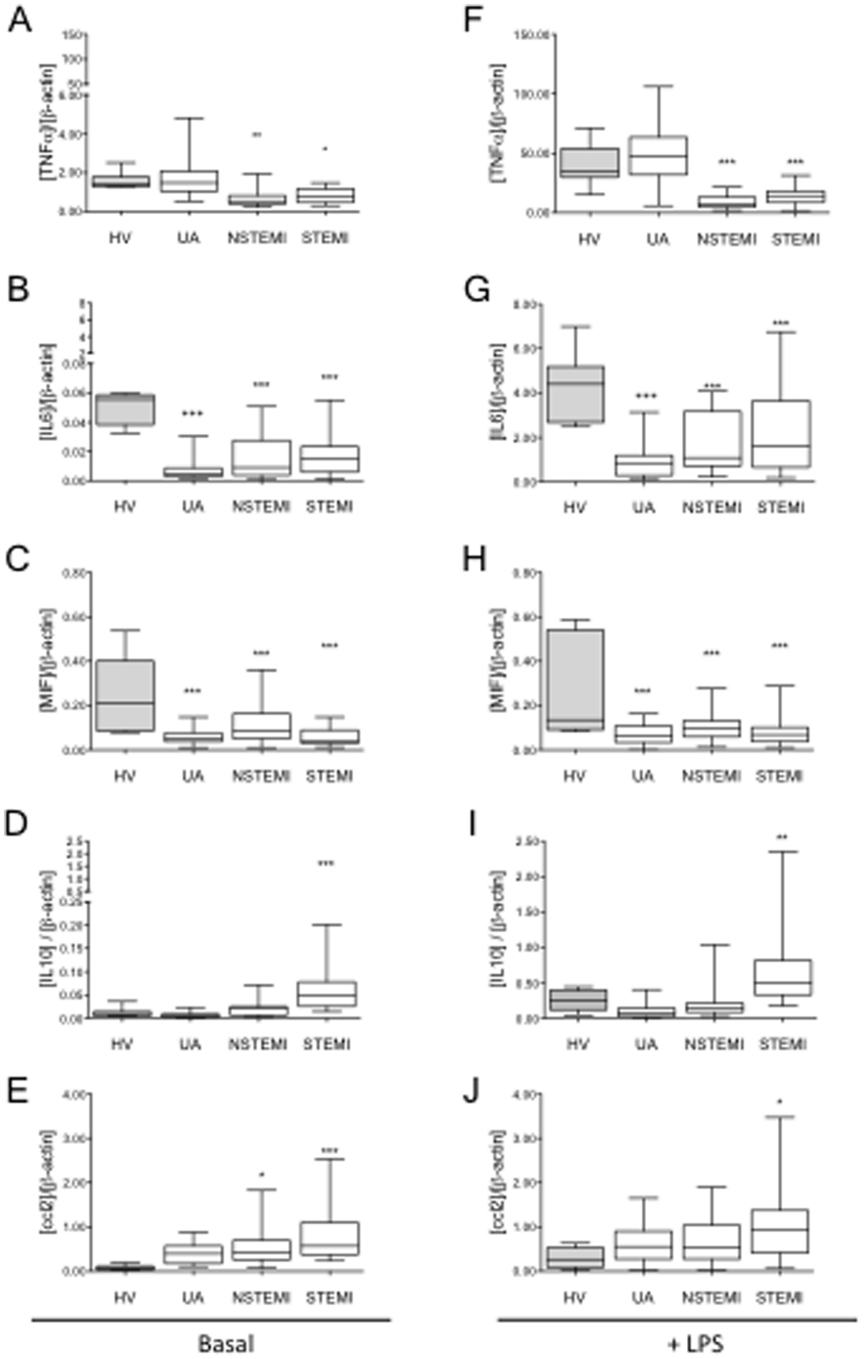
M2 polarization of circulating monocytes in patients with ACS correlates with the severity of the disease. Isolated monocytes from ACS patients were treated (right panel) or not (left panel) with LPS (3 h, 10 ng/ml), total mRNA was isolated and cDNA synthesized. A qPCR analysis of (**A, F**) TNFα, (**B, G**) IL6, (**C, H**) MIF, (**D, I**) IL10 and (**E, J**) CCL2 was performed. Healthy volunteers (HV, gray bars), patients with ACS (white bars). Data are shown as box plots with Mean±SD. HV n = 20, ACS patients: UA n = 29, NSTEMI n =  24, STEMI n = 21; *p<0.05, **p<0.01, ***p<0.001 patients *vs.* HV (ANOVA/Dunn).

Our analysis showed that, whereas the mtDNA in patients with NSTEMI had increased slightly, in patients with STEMI it was markedly higher (∼3.5 fold) compared to patients with UA and HV (Figure 5). Interestingly, monocytes from NSTEMI and, more markedly, from STEMI patients, showed an M2 phenotype (Fig. 6). Whereas the M2 markers IL10 and CCL2 were up-regulated in basal and LPS-challenged monocytes from STEMI, and less markedly in NSTEMI patients, the expression of TNFα was significantly lower in both STEMI and NSTEMI patients. However, in this context we detected no significant changes in TGFβ expression.

**Figure 7:**
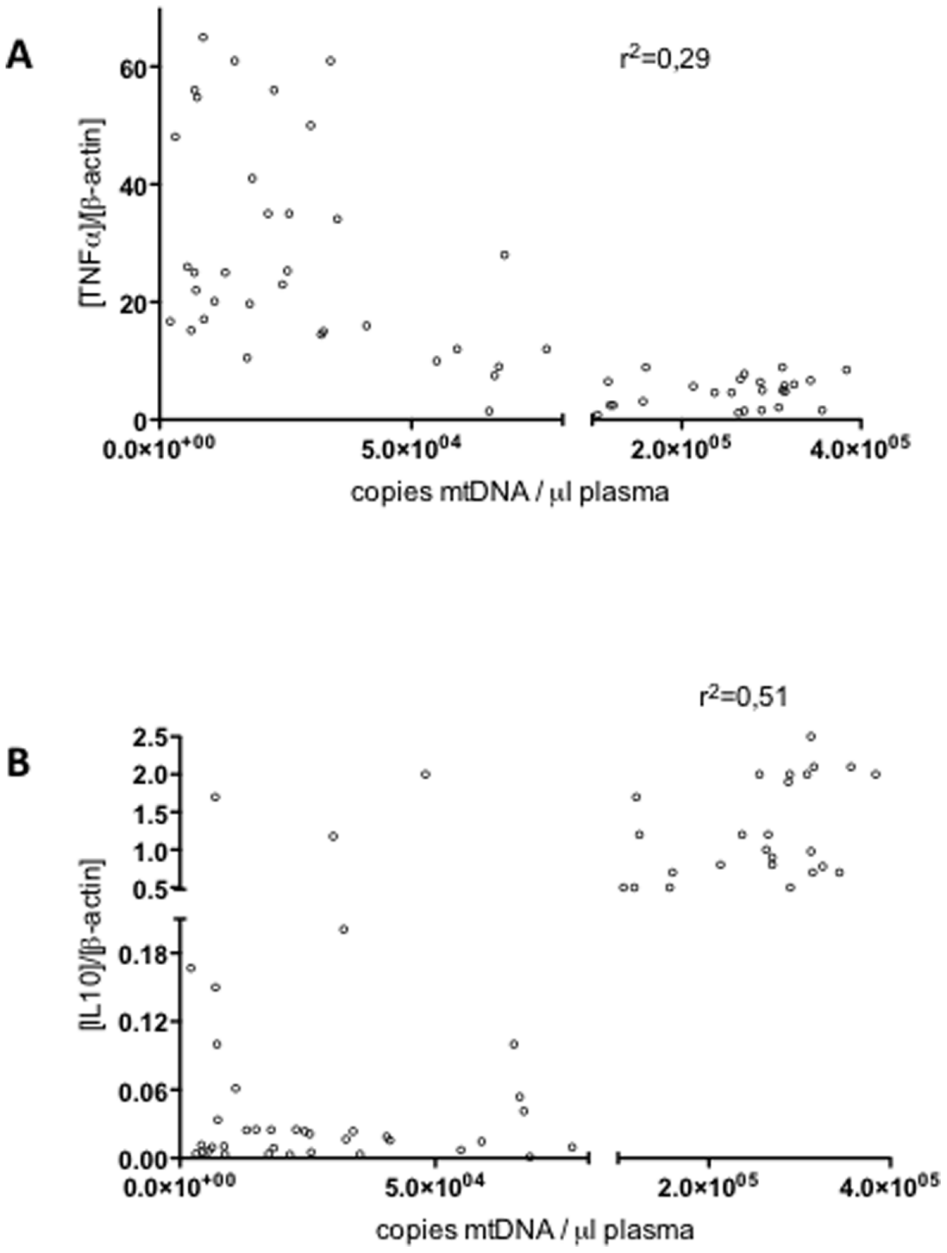
Levels of mtDNA in ACS patients' plasma and cytokine expression. Correlation between plasma mtDNA and TNFα after LPS challenge (3 h, 10 ng/ml, **A**) and IL10 mRNA basal levels (**B**) in MΦs from patients with ACS *r*: Spearman correlation coefficients are shown.

Furthermore, we found a negative correlation between plasma mtDNA and TNFα levels after LPS exposure and a positive correlation with IL10 basal levels (Fig. 7). These data confirm that there is a marked increase in circulating mtDNA in patients with multiple coronary lesions, and that this increase correlates with the M2 phenotype.

### High subsequent rate of infection in ACS patients with M2 circulating monocytes

**Figure 8:**
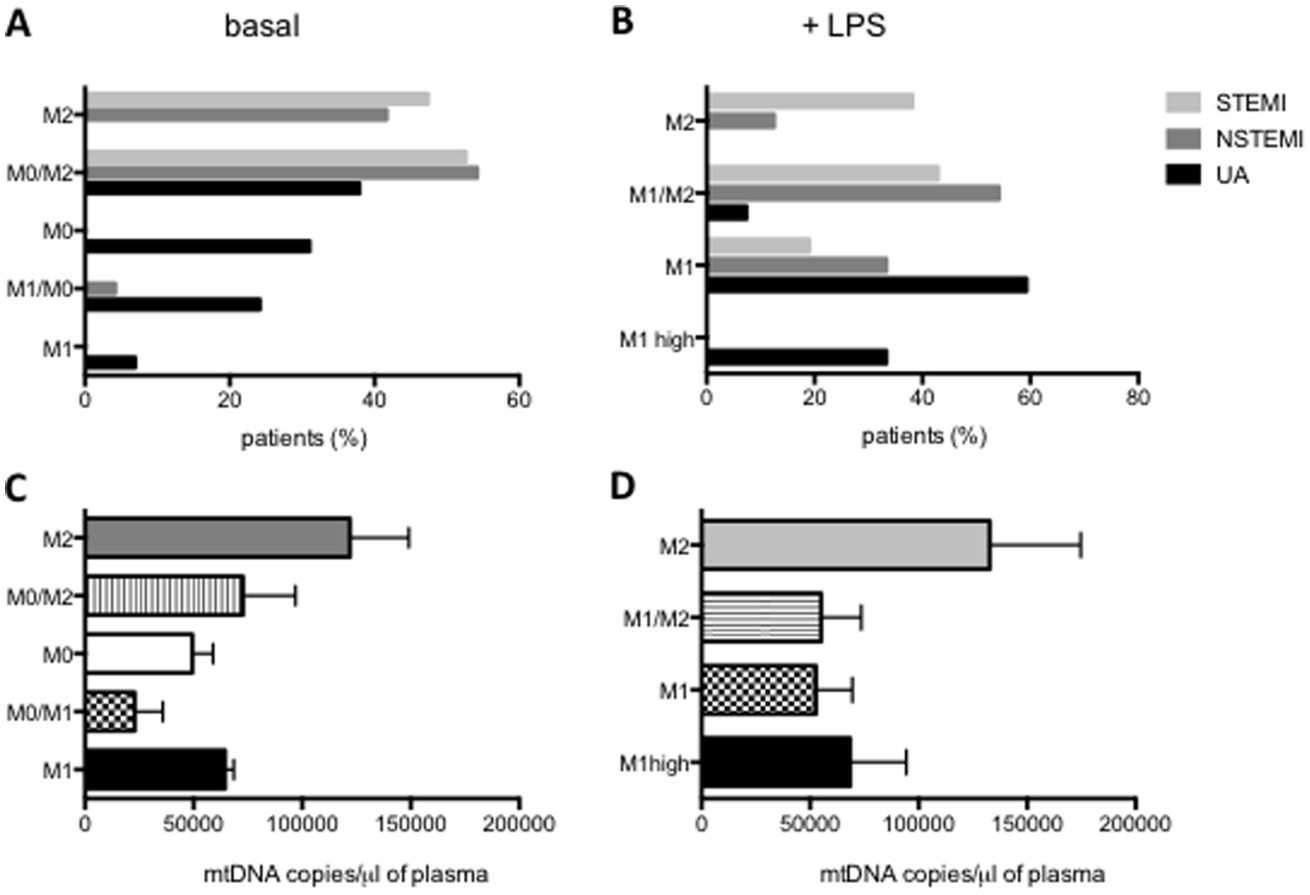
M1/M2 score of ACS patients. (**A–B**) Percentage of patients classified according to the M1/M2 Score of UA (black bars), NSTEMI (dark gray bars) and STEMI (light grey bars) patients, calculated using mRNA expression levels of TNFα and IL10 in MΦs (see Methods). (**A**) At baseline: M1, M1/M0, M0, M0/M2, M2. (**B**) After LPS (3 h, 10 ng/ml): M1high, M1, M1/M2, M2. (**C–D**) Circulating mtDNA levels in the plasma of ACS patients compared with the M1/M2 score. Data are shown as Mean±SD. (**C**) Score at basal condition: M1 n = 2, M1/M0 n = 5, M0 n = 19, M0/M2 n = 28, M2 n = 21. (D) Score after LPS (3 h, 10 ng/ml): M1-high n = 5, M1 n = 27, M1/M2 n = 23, M2 n = 20.

The M2 polarization observed in MΦs from patients with NSTEMI and, more distinctly, in patients with STEMI, could lead to a higher frequency of infection, as seen in other pathologies such as sepsis [1]. To better classify the inflammatory phenotype of the circulating MΦs of patients with ACS, we established an M1/M2 score. The score was calculated using TNFα and IL10 mRNA levels at baseline (Fig. 8A) and after an LPS challenge (Fig. 8B), as we detailed in Material and Methods. At baseline, the MΦs from all the patients with STEMI and most of the patients with NSTEMI were distributed along the M2 score, whereas patients with UA ranged from pro-inflammatory M1 to M0, and to the intermediate state M0/M2 (Fig. 8A). After LPS stimulation, some patients with NSTEMI and STEMI showed a classic M1 phenotype, but they primarily demonstrated an M2 response (Fig. 8B). In contrast, nearly all patients with UA showed a classic M1 response, and some had a stronger M1 (M1 high) response (Fig. 8B). Consistent with these results, the levels of mtDNA were higher in patients with an M2 phenotype (Fig. 8C and 8D). We also carried out a follow-up study of all the ACS patients enrolled in this study. Ten percent of the patients included were re-admitted to the hospital with an infection during the 2 months following enrollment. All of these cases were classified as STEMI (62.5%) or NSTEMI (37.5%). Interestingly, all the infected patients showed an M2 (71.4%) or M0/M2 (28.6%) phenotype according to our score. This observation will need in-depth study to reach a statistically significant conclusion.

## Discussion

Our data indicate that exposure to mitochondrial DAMPs suffices to induce endotoxin tolerance in human monocytes. The production of several pro-inflammatory cytokines was impaired when mtLys-pretreated cultures were challenged with LPS. In contrast, TGFβ levels increased as did the cell surface expression of CD163; both are well known markers of the ET or M2 phenotype [26]. We also observed the expression of a very important negative regulator of the TLR-NFκB pathway: IRAK-M. This pseudokinase is one of the genes that is consistently induced into ET [1], [27]. Here we verified that IRAK-M is up-regulated in LPS-treated monocytes preconditioned with mtLys. In line with these findings, the analysis of p65 translocation into the nucleus revealed an inhibition of this process in mtLys-pretreated cultures. Subsequently, the transcription of pro-inflammatory cytokines was also impaired. Strict controls were established in our assays to ensure the absence of endotoxin traces. All these features prompted us to postulate that mitochondrial DAMPs provoke a refractory state in human monocytes that match the M2 phenotype. Similar results were obtained when mtLys mtDNA, not nuDNA, were used in our *in vitro* model.

In most cases, ET has been associated with previous endotoxin contact, which induces a refractory state in a second endotoxin challenge [4], [18], [28]. These data now indicate that endogenous ligands activate TLR signaling and induce endotoxin tolerance-like features or an M2 phenotype [29], [30]. Therefore, DAMPs play a crucial role in the development of inflammation and the refractory state observed in “sterile” pathologies [11], [29]. Published data suggest that mitochondria can also be a source of DAMPs, as they carry bacterial motifs due to their endosymbiotic origin (i.e., their DNA is rich in CpG motifs and molecules such as cardiolipin in their membrane) [31]–[33]. Our *in vitro* data suggest that during MΦs exposure, mitochondrial DAMPs could be recognized by innate immunity using pattern recognition receptors that alternatively sense bacteria. This might explain why responses to these mitochondrial DAMPs can mimic sepsis and, under sterile conditions, MΦs can develop into an ET state.

To explore the pathophysiological implications of our data, we studied monocytes from ACS patients. We previously reported an early increase in IRAK-M expression in circulating cells from ACS patients without any infection history [3]. Therefore, we hypothesized that IRAK-M induction could be due to the presence of mitochondrial DAMPs and, subsequently, explain a manifest ET status of these patients hours after they were diagnosed. In this regard, our findings suggest that after an ischemic injury, patients shift toward an alternative response and could be more prone to subsequent infections. It has been established that markers of inflammation increase in patients with ACS. However, little is known regarding the potential counterbalancing role of the M2 polarization of MΦs. Our data demonstrated that MΦs from patients with STEMI and NSTEMI showed an M2 phenotype 30 h after MI. Stimulation with LPS can induce an M1 or M2 response, depending on the activation potential of the MΦs. In this context, we observed that MΦs from MI patients not only shifted toward anti-inflammatory M2 activation, but also showed either hypo-responsiveness or ET. Twenty-seven of the 45 patients, all from STEMI and NSTEMI groups, showed MΦs ET after LPS stimulation, whereas none of the 30 patients with UA exhibited ET. Interestingly, the magnitude of the ET differed in patients with STEMI and NSTEMI and correlated with plasma mtDNA concentrations. Symptoms in patients with UA frequently result from an incomplete or transient obstruction of flow in the culprit coronary artery. Consequently, the magnitude of the myocardial ischemic injury is insufficient to cause a release of mtDNA from damaged cells. In contrast, patients with STEMI frequently have an occlusive thrombus complicating a high-grade stenosis, resulting in a complete arrest of flow and extensive myocardial necrosis that release mtDNA from dead cells into the blood circulation.

This type of ET has been widely studied in other pathologies, such as sepsis and cystic fibrosis [4], [10], [34], [35]. Moreover, several authors have demonstrated that increases in plasma of both nuclear and mitochondrial DNA occur in a variety of critical conditions, including sepsis [36], bacterial meningitis [37], trauma [32] and cardiac arrest [38]. We have confirmed a significant increase in circulating mtDNA in patients with a high number of coronary lesions (STEMI). In addition, levels of plasma mtDNA correlate with the M2 phenotype observations of MΦs. Collectively, these data indicate that by acting as DAMPs, mtDNA contributes to inducing an ET state.

Nevertheless, while ET has been considered a protective mechanism against additional damage by the immune system, its incidence is also associated with a high risk of secondary infection [39]. For example, in sepsis, mortality due to secondary infection is associated with the induction of a tolerant state [34]. Similarly, in acute pulmonary syndromes and cystic fibrosis, ET relates to an increased susceptibility to nosocomial infections [35]. Consistent with the ET state of their MΦs, ten percent of all the patients were re-admitted to the hospital with an infection within 2 months following enrollment; all of them were classified as STMEI and NSTEMI. Interestingly, patients who had subsequent infections did not produce TNFα after an LPS challenge, but they did produce IL10 and showed lower levels of pro-inflammatory proteins in plasma than patients with UA. This indicates that, other than MΦs, there could also be a switch of other immune mediators into an anti-inflammatory response. Our score system, based on the most characterized M1 and M2 markers (TNFα and IL10, respectively), showed that MΦs from all patients suffering from a subsequent infection exhibited an evident M2 phenotype. This reveals that after the initial inflammation following cardiac injury that activates innate immune mechanisms, patients shift toward an anti-immune response.

The present data extend our current understanding of endotoxin tolerance implications, not only of endotoxins but also of “sterile” factors such as mitochondrial DAMPs inducing a refractory status in human monocytes. Additionally, our study provides insight into the importance of the innate immune system's state when clinical complications arise after MI. The classification of MΦ's polarization state could be a useful predictor of possible outcomes after cardiac injury, because it is essential for the resolution of inflammation and the transition into the reparative phase. Furthermore, the levels of mitochondrial antigens in plasma, such as plasma mtDNA, should be useful as a marker of increased risk of susceptibility to nosocomial infections in ACS (specifically in STEMI) and in other pathologies where there is tissue damage, such as trauma.

## Supporting Information

Figure S1 **Western blot analysis of IRAK-M.** MΦs from HV were pre-exposed to mitochondrial DNA isolated from HeLa (mtDNA, 5 µg/ml), LPS (10 ng/ml) or left untreated (-) for 5 days, then stimulated for 3 h with LPS. Next, a Western blot analysis of IRAK-M (upper panel) and Actin (lower panel) was performed using total protein extract. A typical blot is shown (n = 2).
(TIFF)

Figure S2 **Western blot analysis of mtLys (three samples) and a nuclear extract (control), nuclear Lamin B (upper panel), mitochondrial Voltage-dependent anion selective channel protein 1/2/3, VDAC1/2/3 (lower panel); a typical blot is shown.**
(TIFF)

Figure S3 **Exposure of Peripheral Blood Mφs to Nuclear DNA.** Peripheral Mφ from healthy volunteers were exposed to nuclear DNA (nuDNA, 5 µg/ml), LPS (10 ng/ml), or left untreated for 5 days and then stimulated 24 h with LPS. Histogram plots of surface HLA-DQ (up) and HLA-DR (down) expression on CD14+ Mφ evaluated by flow cytometry after 24 h of LPS challenge in Mφ pre-exposed 5 days to nuDNA (black line, left panel) or LPS (black line, right panel), compared to Mφ with no pre-treatment (gray filled, all panels). Mean Fold decrease of MFI compared to corresponding control with no pre-treatment.
(TIFF)

Table S1 **CD163+CD14+ frequencies and HLA-DQ, HLA-DR expression in Mφ after mtLys pre-treatment.**
(DOC)

Table S2 **CD163+CD14+ frequencies and HLA-DQ, HLA-DR expression in Mφ after mtDNA and nuDNA pre-treatment.**
(DOCX)

Table S3 **CD163+CD14+ frequencies and HLA-DQ, HLA-DR expression in Mφ after nuDNA pre-treatment.**
(DOCX)

Data S1 **Patient's description.**
(DOCX)
