# Supplementary figures and images for: Mitochondrial DAMPs Induce Endotoxin Tolerance in Human Monocytes: An Observation in Patients with Myocardial Infarction
Source: PLoS One. 2014 May 5;9(5):e95073. doi: 10.1371/journal.pone.0095073 (PMC4010397; doi:10.1371/journal.pone.0095073)

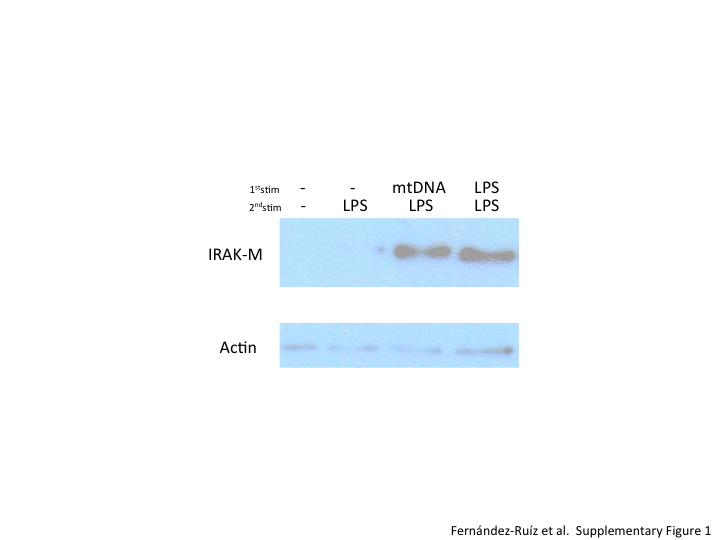

Supplement: Figure S1 — Western blot analysis of IRAK-M. MΦs from HV were pre-exposed to mitochondrial DNA isolated from HeLa (mtDNA, 5 µg/ml), LPS (10 ng/ml) or left untreated (-) for 5 days, then stimulated for 3 h with LPS. Next, a Western blot analysis of IRAK-M (upper panel) and Actin (lower panel) was performed using total protein extract. A typical blot is shown (n = 2). (TIFF) [file pone.0095073.s001.tif]

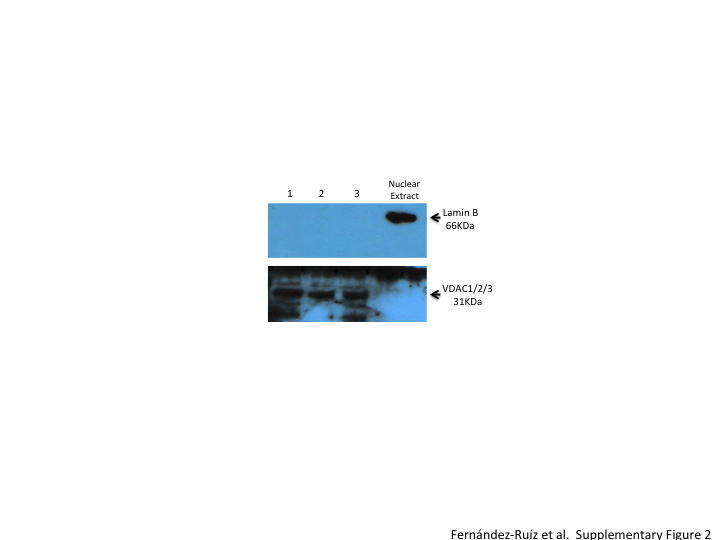

Supplement: Figure S2 — Western blot analysis of mtLys (three samples) and a nuclear extract (control), nuclear Lamin B (upper panel), mitochondrial Voltage-dependent anion selective channel protein 1/2/3, VDAC1/2/3 (lower panel); a typical blot is shown. (TIFF) [file pone.0095073.s002.tif]

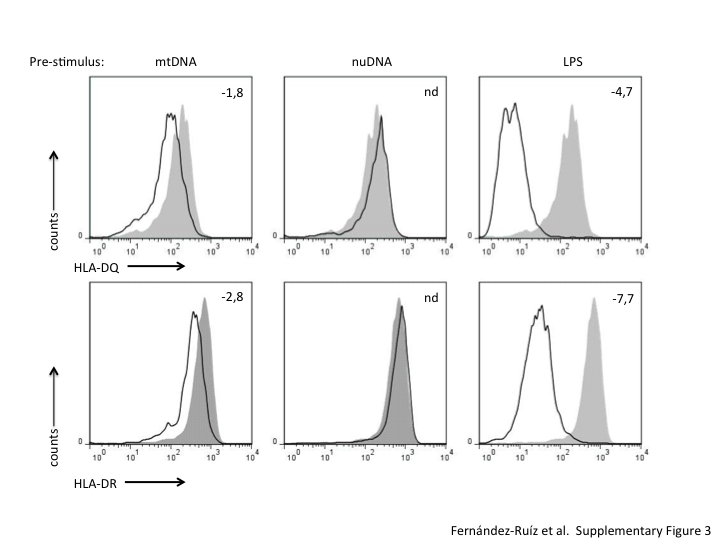

Supplement: Figure S3 — Exposure of Peripheral Blood Mφs to Nuclear DNA. Peripheral Mφ from healthy volunteers were exposed to nuclear DNA (nuDNA, 5 µg/ml), LPS (10 ng/ml), or left untreated for 5 days and then stimulated 24 h with LPS. Histogram plots of surface HLA-DQ (up) and HLA-DR (down) expression on CD14+ Mφ evaluated by flow cytometry after 24 h of LPS challenge in Mφ pre-exposed 5 days to nuDNA (black line, left panel) or LPS (black line, right panel), compared to Mφ with no pre-treatment (gray filled, all panels). Mean Fold decrease of MFI compared to corresponding control with no pre-treatment. (TIFF) [file pone.0095073.s003.tif]
