# Supplementary material for: Mitochondrial DAMPs Induce Endotoxin Tolerance in Human Monocytes: An Observation in Patients with Myocardial Infarction
Source: PLoS One. 2014 May 5;9(5):e95073. doi: 10.1371/journal.pone.0095073 (PMC4010397; doi:10.1371/journal.pone.0095073)
Supplement: Table S1 — CD163+CD14+ frequencies and HLA-DQ, HLA-DR expression in Mφ after mtLys pre-treatment. (DOC) [file pone.0095073.s004.doc]

**Table S1.** CD163+CD14+ frequencies and HLA-DQ, HLA-DR expression in Mφ after mtLys pre-treatment.

| Pre-stimulus: | Medium | mtLys | LPS |
| --- | --- | --- | --- |
| CD163+CD14+ (%) | 6.6 ± 3.7 | 26.2 ± 5.3 ** | 30.8 ± 1.9 *** |
| HLA-DQ (MIF) | 124.8 ± 3.4 | 72.6 ± 8.4 * | 26.7 ± 12.6 *** |
| HLA-DR (MIF) | 224.5 ± 41.8 | 104.6 ± 45.5 ** | 28.9 ± 9.3 *** |

MIF: Mean Intensity Fluorescence. Data are Mean ± SD. *p<0.05, **p<0.01, ***p<0.001 *vs* control of Medium 5d +LPS stimulus.
