# Supplementary material for: Mitochondrial DAMPs Induce Endotoxin Tolerance in Human Monocytes: An Observation in Patients with Myocardial Infarction
Source: PLoS One. 2014 May 5;9(5):e95073. doi: 10.1371/journal.pone.0095073 (PMC4010397; doi:10.1371/journal.pone.0095073)
Supplement: Table S2 — CD163+CD14+ frequencies and HLA-DQ, HLA-DR expression in Mφ after mtDNA and nuDNA pre-treatment. (DOCX) [file pone.0095073.s005.docx]

**Table S2.** CD163+CD14+ frequencies and HLA-DQ, HLA-DR expression in Mφ after mtDNA and nuDNA pre-treatment.

| Pre-stimulus: | Medium | mtDNA | LPS |
| --- | --- | --- | --- |
| CD163+CD14+ (%) | 5.7 ± 2.3 | 9.6 ± 4.1 (ns) | 30.8 ± 1.9 (***) |
| HLA-DQ (MIF) | 124.8 ± 58.7 | 78.3 ± 10.6 (*) | 32.6 ± 22.1 (***) |
| HLA-DR (MIF) | 487.4 ± 297.7 | 173.0 ± 40.0 (*) | 104.1 ± 128.7 (*) |

MIF: Mean Intensity Fluorescence. Data are Mean ± SD. *p<0.05, ***p<0.001, ns: non-significant *vs.* control of Medium 5d +LPS stimulus.
