# Supplementary material for: Mitochondrial DAMPs Induce Endotoxin Tolerance in Human Monocytes: An Observation in Patients with Myocardial Infarction
Source: PLoS One. 2014 May 5;9(5):e95073. doi: 10.1371/journal.pone.0095073 (PMC4010397; doi:10.1371/journal.pone.0095073)
Supplement: Table S3 — CD163+CD14+ frequencies and HLA-DQ, HLA-DR expression in Mφ after nuDNA pre-treatment. (DOCX) [file pone.0095073.s006.docx]

**Table S3.** CD163+CD14+ frequencies and HLA-DQ, HLA-DR expression in Mφ after nuDNA pre-treatment.

| Pre-stimulus: | nuDNA | LPS |
| --- | --- | --- |
| CD163+CD14+ (%) | 16.81 ± 12.15 (ns) | 30.8 ± 1.9 (***) |
| HLA-DQ (MIF) | 129.67 ± 35.67 (ns) | 32.6 ± 22.1 (***) |
| HLA-DR (MIF) | 375.25 ± 179.85 (ns) | 104.1 ± 128.7 (*) |
