# Supplementary material for: Mitochondrial DAMPs Induce Endotoxin Tolerance in Human Monocytes: An Observation in Patients with Myocardial Infarction
Source: PLoS One. 2014 May 5;9(5):e95073. doi: 10.1371/journal.pone.0095073 (PMC4010397; doi:10.1371/journal.pone.0095073)
Supplement: Data S1 — Patient's description. (DOCX) [file pone.0095073.s007.docx]

**Supplementary Methods.**

**Patients**

Patients with UA were defined by the presence of suggestive chest pain at rest occurring during the preceding 6h with transient significant ischemic ST-segment depression or prominent T-waves inversion or both, without significant increases in markers of myocardial necrosis (CK-MB and Troponin cTnI). Patients with AMI were defined according to the joint ESC/ACC 2000 consensus guidelines.[^1^](#_ENREF_1) Non-ST elevation myocardial infarction (NSTEMI) was diagnosed when chest pain typical of myocardial ischemia lasting >20 min was associated with significant elevation of Troponin-I levels (two-fold greater than the upper normal range), with or without ST-segment depression or T wave inversion in the ECG. Acute MI with ST elevation (STEMI) was diagnosed in the presence of typical chest pain longer than 20 min combined with ST-segment elevation in the ECG and typical cTnI rises.[^2^](#_ENREF_2) All enrolled patients were receiving correct treatment for their comorbidities associated with the ACS and, depending on the indications, all of them received standard clinical care for the SCA syndrome according to the guidelines published by the AHA and the ESC.[^3^](#_ENREF_3) All patients were subjected to coronary angiography either on an emergency basis (during hospitalization as part of a scheduled diagnostic work-up). Patients in the three studied groups had a diagnosis of coronary artery disease (CAD) based on coronary angiograms. All patients with STEMI underwent primary percutaneous coronary intervention (PCI) within 12 h after the onset of chest pain.

Selective coronary cineangiography was done from the brachial or femoral approach using Judkin’s or Sones’s technique. Multiple views were obtained in all patients, with the left anterior descending and left circumflex coronary arteries visualized in at least four views and the right coronary artery in at least two views. All coronary and left ventricular angiograms were interpreted by two experienced observers. The degree of stenosis was defined as the greatest percentage reduction of luminal diameter in any view compared with the nearest normal segment (percent diameter stenosis) and was determined using the caliper technique. The vessel score indicated the number of coronary arteries with
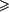
70% stenosis (
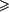
 50% for the left main coronary artery).[^4^](#_ENREF_4) Scores ranged from 0 to 3, depending on the number of vessels involved. Left main coronary artery stenosis was scored as 2-vessel disease. The vessel score was 3 in 6 patients (8%), 2 in 24 (32%), 1 in 31 (41 %), and 0 in 14 (19%). The left main stem was affected in three patients (4%), the left anterior descending coronary artery in 41 (55%), the circumflex coronary artery in 32 (43%), and the right coronary artery in 37 (49%). No significant differences regarding cTnI, or the degree of coronary atherosclerotic severity stenosis were noted between patients with STEMI and NSTEMI.

Plasma BNP was immediately analyzed on the same EDTA-anti-coagulated blood sample collected at admission for troponin-I, using the quantitative immunofluorescence assay manufactured by Biosite (San Diego, USA). The analytic sensitivity of the assay is 0.5 pg/mL and the upper normal limit is considered to be 100 pg/mL. Plasma troponin-I was measured by immunofluorescence assay manufactured by Dade-Behring. The analytic sensitivity of the assay is 0.1 ng/mL and the upper normal limit for the diagnosis of AMI was considered to be 1.0 ng/mL.

**References.**

1. Alpert JS, Thygesen K, Antman E, Bassand JP. Myocardial infarction redefined--a consensus document of the joint european society of cardiology/american college of cardiology committee for the redefinition of myocardial infarction. *J Am Coll Cardiol*. 2000;36:959-969

2. Anderson JL, Adams CD, Antman EM, Bridges CR, Califf RM, Casey DE, Jr., Chavey WE, 2nd, Fesmire FM, Hochman JS, Levin TN, Lincoff AM, Peterson ED, Theroux P, Wenger NK, Wright RS, Smith SC, Jr., Jacobs AK, Halperin JL, Hunt SA, Krumholz HM, Kushner FG, Lytle BW, Nishimura R, Ornato JP, Page RL, Riegel B. Acc/aha 2007 guidelines for the management of patients with unstable angina/non-st-elevation myocardial infarction: A report of the american college of cardiology/american heart association task force on practice guidelines (writing committee to revise the 2002 guidelines for the management of patients with unstable angina/non-st-elevation myocardial infarction) developed in collaboration with the american college of emergency physicians, the society for cardiovascular angiography and interventions, and the society of thoracic surgeons endorsed by the american association of cardiovascular and pulmonary rehabilitation and the society for academic emergency medicine. *J Am Coll Cardiol*. 2007;50:e1-e157

3. O'Connor RE, Bossaert L, Arntz HR, Brooks SC, Diercks D, Feitosa-Filho G, Nolan JP, Vanden Hoek TL, Walters DL, Wong A, Welsford M, Woolfrey K. Part 9: Acute coronary syndromes: 2010 international consensus on cardiopulmonary resuscitation and emergency cardiovascular care science with treatment recommendations. *Circulation*. 2010;122:S422-465

4. Sullivan DR, Marwick TH, Freedman SB. A new method of scoring coronary angiograms to reflect extent of coronary atherosclerosis and improve correlation with major risk factors. *Am Heart J*. 1990;119:1262-1267

5. Andreu AL, Martinez R, Marti R, Garcia-Arumi E. Quantification of mitochondrial DNA copy number: Pre-analytical factors. *Mitochondrion*. 2009;9:242-246

6. Fernandez-Vizarra E, Lopez-Perez MJ, Enriquez JA. Isolation of biogenetically competent mitochondria from mammalian tissues and cultured cells. *Methods*. 2002;26:292-297
